# K-means for shared frailty models

**DOI:** 10.1186/s12874-021-01424-5

**Published:** 2022-01-12

**Authors:** Usha Govindarajulu, Sandeep Bedi

**Affiliations:** Center for Biostatistics, Department of Population Health & Policy Icahn School of Medicine at Mount Sinai, One Gustave Levy Place, NY New York, USA

**Keywords:** Modified k-means algorithm, Shared frailty, Survival analysis, Heterogeneity

## Abstract

**Background:**

The purpose of this research was to see how the k-means algorithm can be applied to survival analysis with single events per subject for defining groups, which can then be modeled in a shared frailty model to further allow the capturing the unmeasured confounding not already explained by the covariates in the model.

**Methods:**

For this purpose we developed our own k-means survival grouping algorithm to handle this approach. We compared a regular shared frailty model with a regular grouping variable and a shared frailty model with a k-means grouping variable in simulations as well as analysis on a real dataset.

**Results:**

We found that in both simulations as well as real data showed that our k-means clustering is no different than the typical frailty clustering even under different situations of varied case rates and censoring. It appeared our k-means algorithm could be a trustworthy mechanism of creating groups from data when no grouping term exists for including in a frailty term in a survival model or comparing to an existing grouping variable available in the current data to use in a frailty model.

## Introduction

The k-means algorithm was designed to find natural groupings amongst bivariate data, essentially creating order from disorder. The method has worked by finding the difference between initial group means and in a process, moving around these means until these distances are minimized. The idea first goes back to Hugo Steinhaus [1] in 1956. He was a Polish mathematician who first came up with the idea as written in his paper. MacQueen also came up with a k-means clustering algorithm by 1967 that is now also used extensively [2] and focused on setting means and finding the centroid of each partition by minimizing sums of squares to the cluster centers, but setting the initial means was never specified. Later on, borne out of signal processing in engineering to partition n observations into k clusters, the first standard algorithm was proposed by Stuart Lloyd of Bells Labs [3]. Lloyd’s work was also known as the Voroni iteration. His iteration focused on finding even set of points in Euclidean partitions and would also would repeatedly find the centroid of each partition and would repeat these operations until finding the centroid that was closet. This type of idea also helped to form the basis behind the k-means algorithm.

The k-means algorithm works to minimize the squared Euclidean distances between clusters. Different variations of this idea have come to fruition over time and several of these proposed algorithms are still used. Even the Expectation-Maximization algorithm was modified in use for this purpose. Of course utilizing different methods of minimizing the distances may produce different results.

In survival analysis, frailty models have allowed incorporating unexplained heterogeneity at the individual level and grouping level [4, 5]. Specifically at the grouping level, there was unmeasured heterogeneity or confounding between groups or clusters of individuals. The models for these were then called shared frailty models. Sometimes, grouping of individuals was available in the data at hand and that natural grouping can be used in the shared frailty model. However, often such term is not available to cluster the individuals but yet, it may suffice that some kind of grouping should be imposed on these individuals in order to model the frailty between them. This idea motivated us to utilize the k-means algorithm to create natural grouping between individuals, which then could be used in a shared frailty model to allow for modeling the heterogeneity between them.

## Methods

### K-means algorithm for survival

In general, the k-means algorithm typically has started with defining number of clusters to partition a priori. In our k-means algorithm for survival, we have allowed making this decision at anywhere between 3 and 5 groups or clusters. Our algorithm then utilized the Euclidean distances between a given pair, x and y, which are randomly chosen existing data points which will become the initial centroids from which to start the algorithm. A typical Euclidean distance formula for the k-means algorithm has been [1–3]: $\mathrm{Distance}=\sqrt{\sum_{\boldsymbol{i}=\mathbf{1}}^{\boldsymbol{n}}{\left({\boldsymbol{x}}_{\boldsymbol{i}}-{\boldsymbol{x}}_{\boldsymbol{c}}\right)}^{\mathbf{2}}+{\left({\boldsymbol{y}}_{\boldsymbol{i}}-{\boldsymbol{y}}_{\boldsymbol{c}}\right)}^{\mathbf{2}}}$***,*** and we modified this formula for the k-means algorithm to be the following for survival data, where we calculate the Euclidean distances between *x* being a main predictor which is continuous and *y* being a survival time, *t*_*i*_, at each ith observation:

1 $$Survival\ Distance=\sqrt{\sum_{i=1}^n{\left({x}_i-{x}_c\right)}^2+{\left({t}_i-{t}_c\right)}^2}$$

We then calculated the distance from each data pair of *x* and survival time for each centroid. Next we sorted the distances and reassign data points to the initial centroids to which they are closest. We then calculated the mean centroid of these groups which became the new centroids for the main continuous predictor and survival time as μ_x_ and μ_t_ respectively. We then repeated the step of calculating the Euclidean distance between each data point pair and the current centroids which starts the loop.

2 $$Updated\ survival\ Distance=\sqrt{\sum_{i=1}^n{\left({x}_i-{\mu}_x\right)}^2+{\left({t}_i-{\mu}_t\right)}^2}$$

We would then calculate this distance and then find the (*x,t*) pair that corresponded to the minimum distance. Those points would then become the new centroids and the step would be repeated over again until the distance between the current centroids and the newly selected centroids became zero. If this step was not obtained in 10 iterations then the loop was completed at 10 iterations and the last set of centroids were then used. These final set of centroids are what help define the grouping variable to be used in a shared frailty model then referred to as a k-means frailty model.

### Cox shared frailty model with smoothing

Survival models, in general, are used to model time to some event. In this application we focus, in particular, on the Cox proportional hazards (PH) regression model with single events per subject. We incorporated a random effect term (frailty), incorporated into a shared frailty model, where frailty was modeled between groups. The frailty term allowed accounting for the unexplained heterogeneity between groups and this was accomplished by specifying a frailty distribution, which is typically modeled by a non-negative one, like a gamma,or log Gaussian distribution. We chose to allow the frailty distribution to have a log-normal distribution for these applications. Using a model as we previously described in a previous manuscript [6], we again incorporated a dichotomous treatment effect as a covariate and again included a continuous, prognostic variable, which then had a smoothing function on it to handle non-linearity as we have previously shown. In a Cox PH regression model this gave [6]:

3 $${h}_{ij}(t)={h}_0(t)\exp \left({x}_{ij}\beta +s\left({y}_{ij}\right)+{w}_i\right)$$

where the following terms are defined as: *h*_*0*_(*t*): baseline hazard rate, *i* = *1,*…,c groups and *j* = *1,*….,*p*_i_ persons per group, *x*_*ij*_: dichotomous treatment variable, *y*_*ij*_: prognostic variable, which was age in our analyses, and *s*(*y*_ij_): smoothed function of *y*_*ij*_. In addition, we again included a log-normal frailty density for the grouping effect [6], similar to Duchateau et al [7]. In Eq.(3), the *i*th cluster was generated as the grouping variable either via the regular method with a variable already available in the dataset to cluster in a shared frailty model or through using our modified k-means clustering previously described procedure to define the groups. These models have been estimated through a penalized regression where the frailty term is treated like an additional covariate but are then penalized by a penalty term added to the log-likelihood according to Therneau et al [8].

From our prior research, we had learned that there are certainly many options for smoothing, and we previously looked extensively at restricted cubic splines, penalized splines, and even fractional polynomials in much detail. However, we again opted to use a natural spline (NS) function as the smoothing function, *s*(*y*_*ij*_), because it is easier to implement and did well in our simulations [9, 10]. We have previously described and implemented in the ns function in R software. We described that natural splines have been essentially restricted cubic splines, and these use B-splines in the basis expansion of *s*(*y*_*ij*_) [6, 9, 10],

4 $$s\left({y}_{ij}\right)={\gamma}_0{y}_{ij}+\sum \limits_{h=1}^{H-2}{\gamma}_h\cdot {B}_h\left({y}_{ij}\right)$$

where *B*_*h*_(*y*_*ij*_) the B-spline basis functions*.* These are described in further detail [9]. We again employed the ns function in R with df = 4, which happens to be the default degrees of freedom (df) in the software. We then compared this shared frailty model from the grouping using our modified k-means algorithm to groupings generated in simulations and we also compared these separate models in a real dataset application with groups defined by a pre-existing grouping variable already available in the dataset. In the next section, we have described our simulations.

### Simulation framework

In this section, we utilized the simulation framework as described in our previous manuscripts [6, 9, 10], where we generated the survival data using methods that similar to those in Bender et al. [11], and described in more detail [9, 12] but adjusted to incorporate a grouping effect in the model. The equation below again represents the true generating model from the Cox PH model [6],

5 $$h\left(t|{x}_{ij},{y}_{ij},{w}_i\right)={h}_0(t)\exp \left({\beta}_T{x}_{ij}+s\left({y}_{ij}\right)+{w}_i\right)$$

As in our previous manuscripts we allowed for the following parameters which we describe in this section [6, 9, 10]. We allowed the subscripts *i* and *j* represent the grouping and person respectively [6]. We again allowed the coefficient *β*_*T*_ to be that for the dichotomous treatment effect *x*_*ij*,_ again, using two treatment scenarios, *β*_*T*_ = 1, and *β*_*T*_ = − 1. We again allowed the s(*y*_*ij*_) = log(*y*_*ij*_) because this was the way to handle any non-linearity in the prognostic factor, *y*_*ij*_. We again sampled *y*_*i j*_ randomly with replacement where we again used an age range of 30 to 66 for the subjects. The grouping effect, *w*_*i*,_ was also again generated from a *N*(0,σ^2^) distribution, with values of *σ*^*2*^ = 0.25 [2].

Again through Bender et al [11] similar to before [6, 9, 10], we again allowed for a baseline Weibull hazard [11, 13], where *h*_0_(*t*) = *θν t*^*θ* ‐ 1^. The survival times end up being found from this generating distribution, when solving that equation for survival time, which as we previously described [6, 9, 10] is using the relationship between the hazard, the survival, and the cumulative distribution functions Also, just like previously, the way this was obtained has been well described in our previous manuscripts [6, 9, 10].

Similar to our previous simulations setups [6, 9, 10], we again included a competing risk into the simulations. We described previously how the competing risk times were generated by employing an exponential distribution to obtain *t*_*cr*_, the competing risk time. Once we did this then we again found the observed survival time to be the minimum of either *t*_*0*_, *t*_*cr*_, and a pre-specified end-of-study time, *τ*, which we put as 20 years as previously described [6, 9, 10]. Also, in order to define right censoring for an observation, we said that an observations was censored if *t*_*0*_ was larger than the minimum of *t*_*cr*_ and *τ*. The final simulated datasets ended up having the treatment varaible, the prognostic factor which was age, the survival times, the event indicators, and then the grouping numbers.

The regular shared frailty model was then compared to a k-means shared frailty model via different parameters through simulations by varying various parameters: number of groups (3, 4, or 5 and an additional 6, 7, or 8), rate of censoring (10% or 70%), case rate (0.0125 or 0.1), coefficient of treatment (− 1 or 1). These variations then led to having 24 possible scenarios and an additional 6 more (25-30) as observed in Table 1. In the comparisons, we used Akaike’s information criterion (AIC) to judge model goodness-of-fit and also the root mean square error (rMSE) [6, 9, 10], which was calculated between the smoothed predictions of the prognostic variable and the observed values of the prognostic variable for a given model in order to assess bias in the predicted and the observed.

**Table 1:** Scenario setup for simulations

***Scenario #***	***Number of groups***	***Censoring %***	***Case rate***	***Treatment coefficient***
*1*	*3*	*10%*	*0.0125*	*trt:-1*
*2*	*3*	*10%*	*0.0125*	*trt: 1*
*3*	*3*	*10%*	*0.1*	*trt: -1*
*4*	*3*	*10%*	*0.1*	*trt: 1*
*5*	*3*	*70%*	*0.0125*	*trt:-1*
*6*	*3*	*70%*	*0.0125*	*trt: 1*
*7*	*3*	*70%*	*0.1*	*trt: -1*
*8*	*3*	*70%*	*0.1*	*trt: 1*
*9*	*4*	*10%*	*0.0125*	*trt:-1*
*10*	*4*	*10%*	*0.0125*	*trt: 1*
*11*	*4*	*10%*	*0.1*	*trt: -1*
*12*	*4*	*10%*	*0.1*	*trt: 1*
*13*	*4*	*70%*	*0.0125*	*trt:-1*
*14*	*4*	*70%*	*0.0125*	*trt: 1*
*15*	*4*	*70%*	*0.1*	*trt: -1*
*16*	*4*	*70%*	*0.1*	*trt: 1*
*17*	*5*	*10%*	*0.0125*	*trt: -1*
*18*	*5*	*10%*	*0.0125*	*trt: 1*
*19*	*5*	*10%*	*0.1*	*trt: -1*
*20*	*5*	*10%*	*0.1*	*trt: 1*
*21*	*5*	*70%*	*0.0125*	*trt:-1*
*22*	*5*	*70%*	*0.0125*	*trt: 1*
*23*	*5*	*70%*	*0.1*	*trt: -1*
*24*	*5*	*70%*	*0.1*	*trt: 1*
*Additional simulations*				
*25*	*6*	*10%*	*0.1*	*trt: 1*
*26*	*6*	*70%*	*0.1*	*trt: 1*
*27*	*7*	*10%*	*0.1*	*trt: 1*
*28*	*7*	*70%*	*0.1*	*trt: 1*
*29*	*8*	*10%*	*0.1*	*trt: 1*
*30*	*8*	*70%*	*0.1*	*trt: 1*

All above programming was done in the R language [14]. We developed our modified k-means algorithm for survival in R and we utilized and conducted all other analyses in R as well.

## Results

### Simulation results

We have shown coefficients, standard errors, and *p*-values from the regular shared frailty model (Table 2) and from the k-means frailty model (Table 3). We have presented the coefficients and *p*-value for treatment (trt), the main predictor. Since a natural spline was fit on age in the model, to summarize this fit, instead of presenting the coefficients from each basis function, we have presented the average *p*-values from amongst the 4 basis functions. We can see that the trt coefficients, standard errors and *p*-values do not differ much between Tables 2 and 3, nor do the average basis coefficient *p*-values. However, we do see that the *p*-values for the frailty variances (Table 4) differs between the models, not initially, however, with increased group size of 5 or greater the frailty variances start showing more statistical significance,, and for the k-means frailty model, which became even smaller in this instance as compared to the regular shared frailty model. Somehow there was more heterogeneity in the k-means frailty models, which is interesting since it works to minimize error..

**Table 2:** Various estimates for regular shared frailty models from simulations

Scenario #	Trt coef regular^**a**^	Trt s.e. regular^**a**^	Trt ***p***-value regular	Average Ns Basis ***p***-value regular
**1**	**NA**	0.3987	NA	0.4311
**2**	1.0266	0.2697	0.0055	0.3827
**3**	−1.0224	0.2074	0.0003	0.3404
**4**	1.0307	0.1817	< 0.0001	0.3050
**5**	−1.0111	0.2324	0.0033	0.3661
**6**	1.0216	0.1608	< 0.0001	0.2650
**7**	−1.0104	0.1303	< 0.0001	0.1910
**8**	1.0139	0.1233	< 0.0001	0.1723
**9**	−1.0467	0.3989	0.0481	0.4218
**10**	1.0391	0.2694	0.0045	0.3758
**11**	−1.0346	0.2070	0.0003	0.3151
**12**	1.0250	0.1810	< 0.0001	0.2775
**13**	−1.0264	0.2309	0.0017	0.3372
**14**	1.0129	0.1599	< 0.0001	0.2535
**15**	−1.0178	0.1304	< 0.0001	0.1853
**16**	1.0145	0.1233	< 0.0001	0.1590
**17**	−1.0471	0.3995	0.0479	0.4427
**18**	1.0424	0.2706	0.0044	0.3785
**19**	−1.0416	0.2076	0.0002	0.3306
**20**	1.0180	0.1808	< 0.0001	0.2908
**21**	−1.0053	0.2303	0.0013	0.3537
**22**	1.0129	0.1600	< 0.0001	0.2653
**23**	−1.0218	0.1309	< 0.0001	0.1928
**24**	1.0126	0.1235	< 0.0001	0.1674
**25**	1.0277	0.1819	< 0.0000	0.2830
**26**	1.0151	0.1239	< 0.0001	0.1650
**27**	1.0108	0.1818	< 0.0001	0.2950
**28**	1.0072	0.1237	< 0.0001	0.1760
**29**	1.0353	0.1825	< 0.0001	0.3000
**30**	1.0057	0.1235	< 0.0001	0.1680

^a^*coef* coefficient, *s.e.* standard error

**Table 3:** Various estimates for k-means shared frailty model from simulations

Scenario #	Trt coef k-means*	Trt s.e. k-means*	Trt ***p***-value k-means	Average Ns Basis ***p***-value k-means
**1**	− 1.0914	324.433	0.0526	0.4314
**2**	1.0202	0.2693	0.0056	0.3829
**3**	−1.0135	0.2069	0.0005	0.3415
**4**	1.0210	0.1812	< 0.0001	0.3078
**5**	−1.0085	0.2323	0.0034	0.3664
**6**	1.0158	0.1606	< 0.0001	0.2653
**7**	−0.9974	0.1299	< 0.0001	0.1950
**8**	1.0030	0.1230	< 0.0001	0.1750
**9**	−1.0442	0.3988	0.0486	0.4220
**10**	1.0350	0.2692	0.0047	0.3781
**11**	−1.0297	0.2067	0.0003	0.3187
**12**	1.0157	0.1807	< 0.0001	0.2778
**13**	−1.0238	0.2307	0.0018	0.3386
**14**	1.0070	0.1598	< 0.0001	0.2563
**15**	−1.0106	0.1302	< 0.0001	0.1583
**16**	1.0050	0.1230	< 0.0001	0.1621
**17**	−1.0445	0.3992	0.0477	0.4439
**18**	1.0381	0.2702	0.0047	0.3806
**19**	−1.0346	0.2072	0.0002	0.3326
**20**	1.0086	0.1803	< 0.0001	0.2935
**21**	−1.0025	0.2301	0.0014	0.3555
**22**	1.0059	0.1598	< 0.0001	0.2655
**23**	−1.0094	0.1304	< 0.0001	0.1953
**24**	0.9981	0.1231	< 0.0001	0.1715
**25**	1.0220	0.1817	< 0.0001	0.2880
**26**	1.0027	0.1234	< 0.0001	< 0.0001
**27**	1.0035	0.1813	< 0.0001	0.1710
**28**	0.9953	0.1232	< 0.0001	0.3010
**29**	1.0253	0.1818	< 0.0001	0.1810
**30**	0.9934	0.1231	< 0.0001	0.3010

^*^coef coefficient, *s.e.* standard error

**Table 4:** Mean MSE and AIC for regular & k-means shared frailty models from simulations

Scenario #	Frailty ***p***-value regular	Frailty ***p***-value k-means	AIC regular	AIC k-means	MSE regular	MSE k-means
**1**	0.4311	0.4708	302.093	302.404	6.087	6.059
**2**	0.3412	0.3838	589.745	590.649	4.450	4.428
**3**	0.2709	0.3356	978.151	979.739	4.060	4.039
**4**	0.2320	0.3120	1318.412	1320.681	3.843	3.849
**5**	0.3013	0.3581	990.889	992.096	4.079	4.072
**6**	0.1990	0.2890	1855.438	1858.139	3.590	3.589
**7**	0.1410	0.2260	2727.565	2732.042	3.578	3.601
**8**	0.1300	0.2010	3135.058	3139.699	3.742	3.754
**9**	0.4021	0.4166	304.075	304.314	6.064	6.032
**10**	0.2976	0.3435	593.701	594.367	4.346	4.351
**11**	0.2312	0.2883	984.750	986.094	3.827	3.855
**12**	0.1960	0.2400	1324.327	1326.034	3.725	3.728
**13**	0.2675	0.3366	1005.633	1006.984	4.083	4.092
**14**	0.1790	0.2310	1875.053	1877.312	3.619	3.631
**15**	0.1270	0.1730	2740.402	2743.620	3.576	3.583
**16**	0.1090	0.1610	3149.836	3153.604	3.555	3.564
**17**	0.3759	0.4107	301.481	301.947	5.931	5.933
**18**	0.3058	0.3521	588.084	588.830	4.454	4.450
**19**	0.2146	0.2817	983.022	984.564	4.117	4.136
**20**	0.2040	0.2700	1330.645	1332.710	3.938	3.960
**21**	0.2531	0.3175	997.525	998.560	4.136	4.154
**22**	0.1820	0.2510	1879.733	1882.331	3.796	3.806
**23**	0.1100	0.1770	2732.218	736.838	3.663	3.681
**24**	0.0900	0.1490	3148.873	3154.325	3.627	3.645
**25**	0.0578	0.0407	1325.762	1327.56	3.637	3.664
**26**	0.0560	0.0255	3145.217	3150.437	3.629	3.645
**27**	0.0614	0.0379	1321.419	1323.576	3.813	3.831
**28**	0.0519	0.0254	3144.961	3149.621	3.663	3.701
**29**	0.0650	0.0420	1326.753	1328.96	3.986	3.992
**30**	0.0582	0.0318	3155.085	3160.036	3.637	3.659

In Table 4, we also have laid out side by side comparisons of the AIC and MSE of each scenario compared between the regular shared frailty model and the k-means shared frailty model. We have seen the goodness-of-fit and the bias appear similar between the two different models for a given scenario for the most part. Though the groupings used in the frailty term in the separate Cox models were derived from different processes, k-means vs simulated grouping creation, the results showed many similarities between the two processes.

**Fig. 1:**
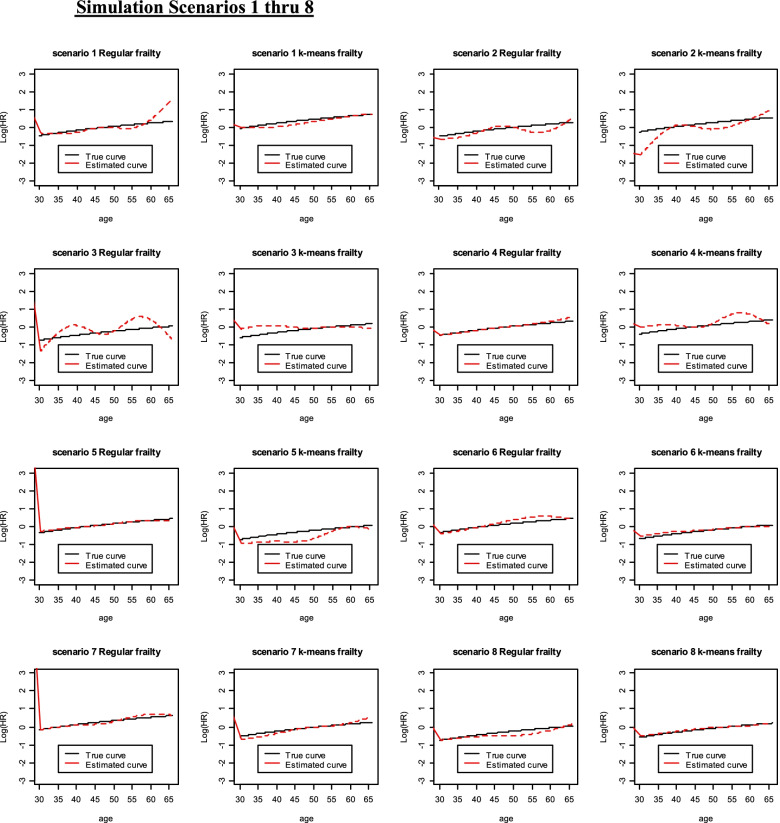
Simulation Scenarios 1 thru 8

**Fig. 2:**
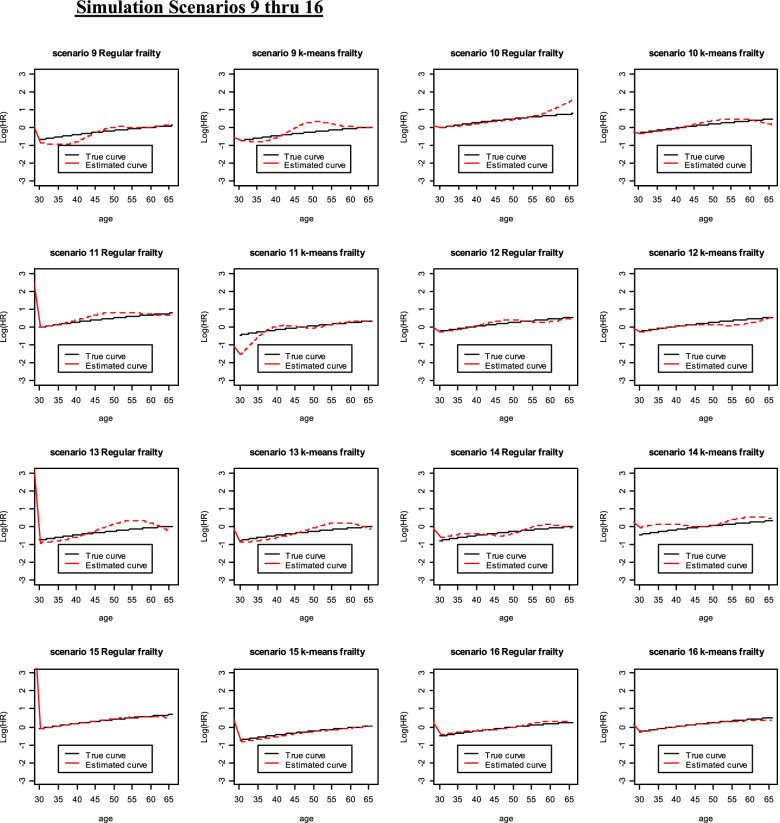
Simulation Scenarios 9 thru 16

**Fig. 3:**
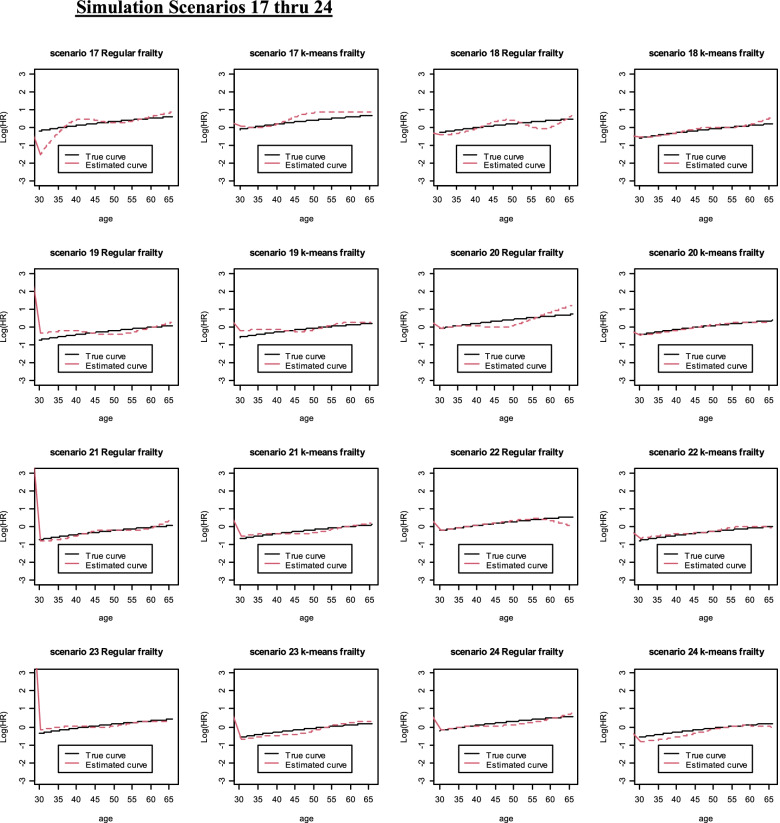
Simulation Scenarios 17 thru 24

**Fig. 4:**
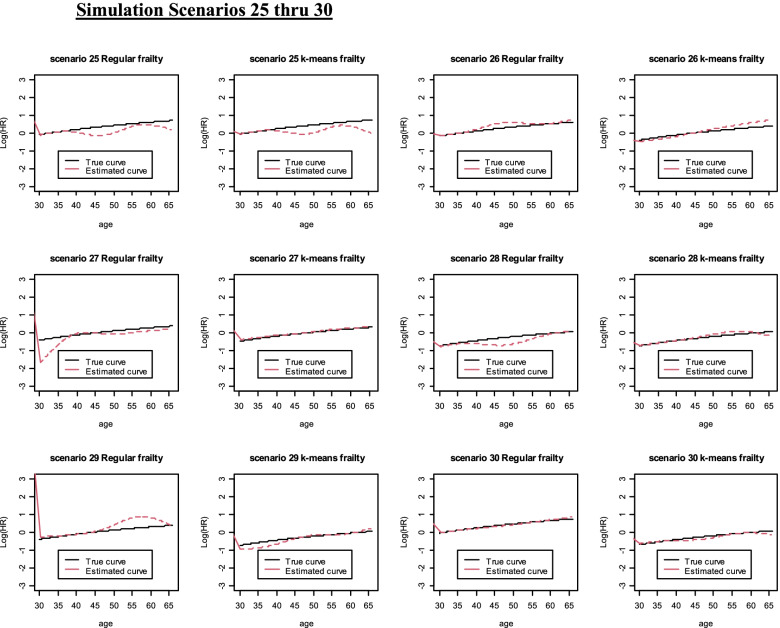
Simulation Scenarios 25 thru 30

Higher case rate but more censoring seems to have led to a better model fit according to MSE which improved with increased group size while the AIC reflected better model fit for lower censoring and lower case rate. However, in general, the model fits for the regular frailty model or for the k-means frailty model compared to its true curve per scenario did not differ much as seen in Figs. 1, 2, 3 and 4. This reflects what has been seen in Table 4 with the MSE and AIC values for both models per scenario.

### Real dataset example

**Fig. 5:**
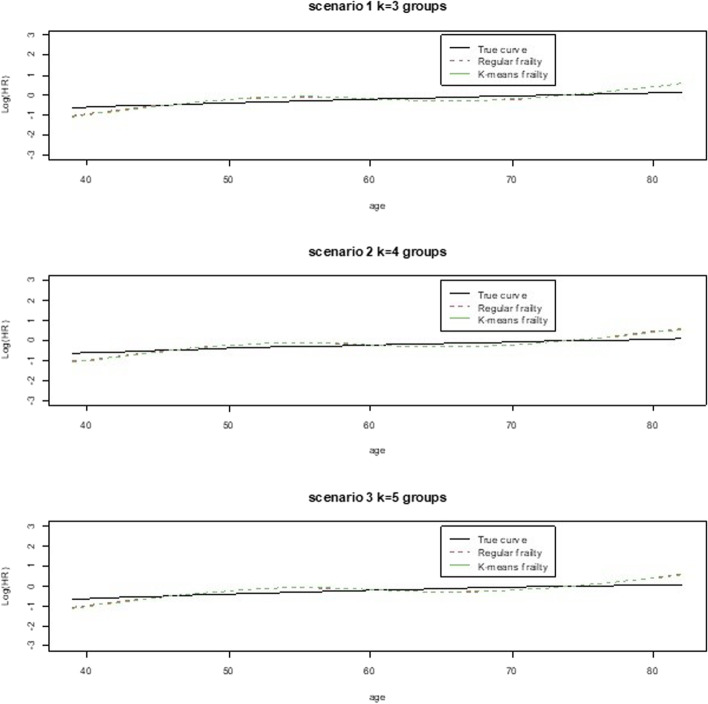
Real dataset plots for estimated log(HR) for truth compared to model fits by number of groups: 3-5

**Fig. 6:**
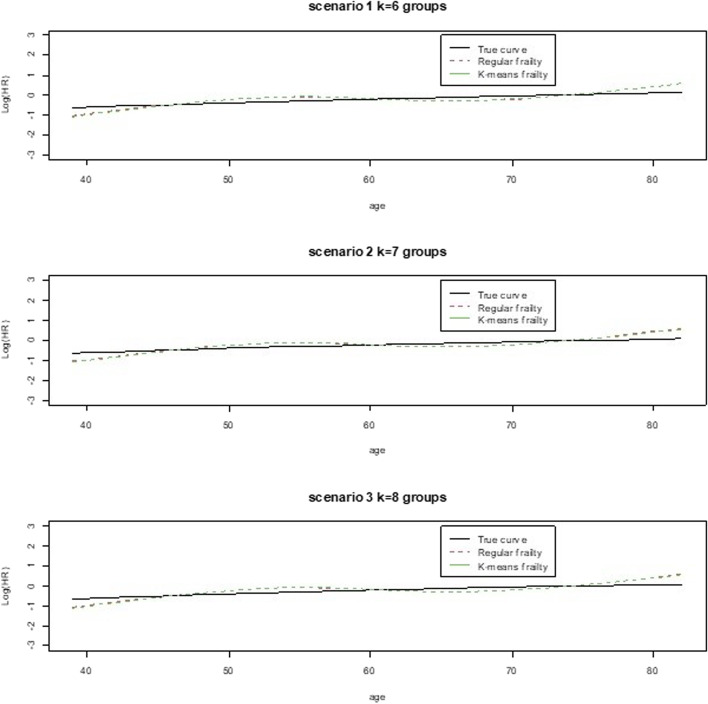
Real dataset plots for estimated log(HR) for truth compared to model fits by number of groups: 6 - 8

We used a lung cancer dataset, which is freely available through the R software [14] in their available datasets in the library, survival. This dataset contained survival data in patients with advanced lung cancer from the North Central Cancer Treatment Group [15]. Performance scores rated how well the patient can perform usual daily activities. We were able to model a shared frailty model using the grouping variable, institution, and a k-means frailty model with grouping provided by our modified k-means algorithm. For both models we ran them for k = 3,4,5,6,7 or 8 groups, to match groupings used in the simulations. We can see in Tables 5 and 6, no differences in treatment coefficients between groups or between models and very minor differences in basis coefficients between groups and models. Various estimates computed for these models and groups have further reflected minor to no differences between the methods, similar to the simulations (Table 6), however there did appear to be more heterogeneity in the regular shared model than the k-means shared model as reflected through the frailty variance. This was opposite of what we had seen in the simulations but seems more consistent with how the k-means works in minimizing variability to create groupings. In addition, the AIC reflected a somewhere better fit for the regular shared frailty models while the MSE reflected a closer fit between observed and predicted for the k-means than the regular shared frailty model. The graphs for the log(HR)‘s of the prognostic factor, age, in Figs. 5 and 6 also reflected the closer fits between the observed age and the predicted HRs from the two different frailty models. All curves were overlapping for each cluster size, 3-8.

**Table 5:** Parameter estimates and measures of fit from lung dataset example for regular and k-means shared frailty models for 3 -5 groups

	**Coefficient regular**			**Coefficient k-means**		
**Parameter**	**3 groups**	**4 groups**	**5 groups**	**3 groups**	**4 groups**	**5 groups**
**Trt**	0.4718	0.4718	0.4718	0.4512	0.4511	0.4509
**Ns basis 1**	0.7456	0.7456	0.7456	0.7769	0.7789	0.7779
**Ns basis 2**	0.3552	0.3552	0.3552	0.3829	0.3882	0.3864
**Ns basis 3**	2.3404	2.3404	2.3404	2.4377	2.4404	2.4396
**Ns basis 4**	1.0204	1.0204	1.0204	1.0046	1.0001	1.002
	**Standard error regular**			**Standard error k-means**		
**Parameter**	**3 groups**	**4 groups**	**5 groups**	**3 groups**	**4 groups**	**5 groups**
**Trt**	0.1201	0.1201	0.1201	0.1174	0.1174	0.1175
**Ns basis 1**	0.6215	0.6215	0.6215	0.6212	0.6210	0.6210
**Ns basis 2**	0.5182	0.5182	0.5182	0.5140	0.5140	0.5138
**Ns basis 3**	1.5051	1.5051	1.5051	1.5028	1.5023	1.5028
**Ns basis 4**	0.6395	0.6395	0.6395	0.6342	0.6349	0.6349
	***p*-values regular**			***p*-values k-means**		
**Parameter**	**3 groups**	**4 groups**	**5 groups**	**3 groups**	**4 groups**	**5 groups**
**Trt**	< 0.0001	< 0.0001	< 0.0001	< 0.0001	< 0.0001	< 0.0001
**Ns basis 1**	0.2303	0.2303	0.2303	0.2111	0.2098	0.2103
**Ns basis 2**	0.4931	0.4931	0.4931	0.4562	0.4501	0.452
**Ns basis 3**	0.1200	0.1200	0.1200	0.1048	0.1043	0.1045
**Ns basis 4**	0.1105	0.1105	0.1105	0.1132	0.1152	0.1145
**frailty**	0.3029	0.3029	0.3029	0.5665	0.4805	0.5003
***p*-values for non-linearity**	0.0003	0.0030	0.0003	0.2046	0.1956	0.1981
**AIC**	1466.74	1466.74	1466.74	1477.51	1477.44	1477.49
**MSE**	2.13	2.13	2.13	1.91	1.90	1.90
**Frailty variance**	0.017	0.017	0.017	0.0007	0.0007	0.0007
**Mean time**	305.23

**Table 6:** Parameter estimates and measures of fit from lung dataset example for regular and k-means shared frailty models for 6 -8 groups

	**Coefficient regular**			**Coefficient k-means**		
**Parameter**	**6 groups**	**7 groups**	**8 groups**	**6 groups**	**7 groups**	**8 groups**
**Trt**	0.4718	0.4718	0.4718	0.4511	0.4524	0.4511
**Ns basis 1**	0.7456	0.7456	0.7456	0.7764	0.7774	0.7802
**Ns basis 2**	0.3552	0.3552	0.3552	0.3843	0.3826	0.3872
**Ns basis 3**	2.3404	2.3404	2.3404	2.4412	2.4460	2.4460
**Ns basis 4**	1.0204	1.0204	1.0204	1.0055	1.0088	1.0088
	**Standard error regular**			**Standard error k-means**		
**Parameter**	**6 groups**	**7 groups**	**8 groups**	**6 groups**	**7 groups**	**8 groups**
**Trt**	0.1201	0.1201	0.1201	0.1174	0.1176	0.1174
**Ns basis 1**	0.6215	0.6215	0.6215	0.6211	0.6212	0.6211
**Ns basis 2**	0.5182	0.5182	0.5182	0.5138	0.5137	0.5136
**Ns basis 3**	1.5051	1.5051	1.5051	1.5038	1.5037	1.5028
**Ns basis 4**	0.6395	0.6395	0.6395	0.6353	0.6360	0.6353
	***p*-values regular**			***p*-values k-means**		
**Parameter**	**6 groups**	**7 groups**	**5 groups**	**3 groups**	**4 groups**	**5 groups**
**Trt**	< 0.0001	< 0.0001	< 0.0001	0.0001	0.0001	0.0001
**Ns basis 1**	0.2303	0.2303	0.2303	0.2113	0.2108	0.2091
**Ns basis 2**	0.4931	0.4931	0.4931	0.4545	0.4563	0.4510
**Ns basis 3**	0.1200	0.1200	0.1200	0.1045	0.1047	0.1036
**Ns basis 4**	0.1105	0.1105	0.1105	0.1135	0.1127	0.1124
***p*-values for non-linearity**	0.0003	0.0003	0.0003	0.2047	0.1940	0.1865
**AIC**	1466.74	1466.74	1466.74	1477.58	1477.43	1477.37
**MSE**	2.13	2.13	2.13	1.90	1.90	1.90
**Frailty variance**	0.017	0.017	0.017	0.0007	0.0007	0.0007
**Mean time**	305.23

## Discussion

Through our research, we attempted to bridge a gap between unsupervised learning and statistical modeling of time-to-event data by combining the k-means algorithm with survival models, namely shared frailty models. We first created our own version of the k-means algorithm by adapting it to use survival time and one main continuous predictor. We then demonstrated comparing a regular shared frailty model with a regular grouping variable and a shared frailty model with a grouping variable created through our modified k-means algorithm for survival. We did this first through simulations and then on analysis of a real dataset. We found that our modified k-means clustering appeared no different than the typical frailty clustering even under different situations of varied case rates and censoring and perhaps had created groups that had roughly the same or less amount of heterogeneity between groups.. Therefore our modified k-means algorithm could be employed as a mechanism of creating groups from data for including in a frailty term in a survival model when there is no grouping variable available, which is the case in many survival datasets, or for comparing to an existing grouping variable used in a frailty model. Some limitation may be to know how many groups or clusters to create but one can always conduct several iterations of using our k-means algorithm for such purpose and then decide using mean-squared errors and information criterion, similar to our analyses.

## Conclusions

We were able to demonstrate that through our modified k-means algorithm for survival data that our k-means approach for survival data could be used to create groupings in data where there was no pre-existing grouping variable, and therefore, this grouping terms could be implemented in a shared frailty model setting to capture unexplained heterogeneity not captured by covariates. We had compared this with a regular grouping term in simulated data as well as a real dataset and found there were no significant differences between our approach and the more conventional approach. Therefore, we recommend use for our approach when an investigator would like to implement a frailty model with survival data but do not have a clear grouping term available in the data to run such a model.

## Data Availability

Dataset is available in the R software. All data generated or analyzed during this study are included in Loprinzi et al (see References).

## References

[1] Steinhaus H (1956). Sur la division des corp materiels en parties. Bull Acad Polon Sci.

[2] MacQueen JB. Some methods for classification and analysis of multivariate observations, vol. 1. Berkeley: University of California Press; 1967. p. 281–97.

[3] LLoyd SP (1982). Least squares quantization in PCM. IEEE Trans Inf Theory.

[4] Govindarajulu US, D'Agostino Sr. RB. Review of current advances in survival analysis and frailty models. WIREs Comput Stat. 2020;12:e1504. 10.1002/wics.1504.

[5] Govindarajulu US, Lin H, Lunetta KL, D'Agostino RB (2011). Frailty models: applications to biomedical and genetic studies. Stat Med.

[6] Govindarajulu US, Malloy EJ (2015). Evaluating treatment effect in multicenter trials with small centers using survival modeling. Int J Stat Med Res.

[7] Duchateau L, Janssen P, Lindsey P, Legrand C, Nguti R, Sylvester R. The shared frailty model and the power for heterogeneity tests in multicenter trials. Comput Stat Data Anal. 2002;40(30);603–20.

[8] Therneau T, Grambsch P, Pankratz V (2003). Penalized survival models and frailty. J Comput Graph Stat.

[9] Govindarajulu US, Malloy EJ, Ganguli B, Spiegelman D, Eisen EA (2009). The comparison of alternative smoothing methods for fitting non-linear exposure-response relationships with Cox models in a simulation study. Int J Biostat.

[10] Govindarajulu US, Spiegelman D, Thurston SW, Ganguli B, Eisen EA (2007). Comparing smoothing techniques in cox models for exposure-response relationships. Stat Med.

[11] Bender R, Augustin T, Blettner M (2005). Generating survival times to simulate cox proportional hazards models. Stat Med.

[12] Malloy EJ, Spiegelman D, Eisen EA (2009). Comparing measures of model selection for penalized splines in cox models. Comput Stat Data Anal.

[13] Klein JM, M. (1997). Survival analysis: techniques for censored and truncated data.

[14] R Core Team (2020). R: A language and environment for statistical computing. R Foundation for Statistical Computing, Vienna, Austria. URL https://www.R-project.org/.

[15] Loprinzi CL, Laurie JA, Wieand HS (1994). Prospective evaluation of prognostic variables from patient-completed questionnaires. North central Cancer treatment group. J Clin Oncol.

